# Butterfly eyespot organiser: *in vivo* imaging of the prospective focal cells in pupal wing tissues

**DOI:** 10.1038/srep40705

**Published:** 2017-01-17

**Authors:** Mayo Iwasaki, Yoshikazu Ohno, Joji M. Otaki

**Affiliations:** 1 The BCPH Unit of Molecular Physiology, Department of Chemistry, Biology and Marine Science, University of the Ryukyus, Okinawa 903-0213, Japan

## Abstract

Butterfly wing eyespot patterns are determined in pupal tissues by organisers located at the centre of the prospective eyespots. Nevertheless, organiser cells have not been examined cytochemically *in vivo*, partly due to technical difficulties. Here, we directly observed organiser cells in pupal forewing epithelium via an *in vivo* confocal fluorescent imaging technique, using 1-h post-pupation pupae of the blue pansy butterfly, *Junonia orithya*. The prospective eyespot centre was indented from the plane of the ventral tissue surface. Three-dimensional reconstruction images revealed that the apical portion of “focal cells” at the bottom of the eyespot indentation contained many mitochondria. The mitochondrial portion was connected with a “cell body” containing a nucleus. Most focal cells had globular nuclei and were vertically elongated, but cells in the wing basal region had flattened nuclei and were tilted toward the distal direction. Epithelial cells in any wing region had cytoneme-like horizontal processes. From 1 h to 10 h post-pupation, nuclear volume increased, suggesting DNA synthesis during this period. Morphological differences among cells in different regions may suggest that organiser cells are developmentally ahead of cells in other regions and that position-dependent heterochronic development is a general mechanism for constructing colour patterns in butterfly wings.

Functional characterisation of organisers has been a central theme of developmental biology since the discovery of the Spemann-Mangold organiser, through transplantation experiments, in amphibian early embryogenesis^1^,^2^. An organiser is a cluster of cells that can induce differentiation of their surrounding cells, and many molecules that are critically involved in the induction process have been identified^1^,^2^. Their molecular network is fairly complex. However, a theoretical approach to the induction process is based on the assumption that organiser cells secrete a putative substance called a morphogen^3^,^4^. Pluripotent cells obtain “positional information” as a specific concentration level of morphogen released from organisers. The fate determination is made if the received concentration exceeds an inherent threshold that is established in the cell. This model has been known as the gradient model for positional information.

Butterfly eyespot colour patterns have been considered to be an excellent system that can be explained well by the classical gradient model for positional information^5^,^6^. This is because a “typical” butterfly eyespot is composed of concentric rings of various colours in a two-dimensional plane, which is reminiscent of diffusional morphogen propagation from the centre of an eyespot. Importantly, based on the following two points, the prospective eyespot centre in the pupal wing tissue indeed functions as an organiser for the eyespot. First, surrounding cells fail to produce eyespot patterns of normal size when the central cells of the prospective eyespot are physically damaged^7^,^8^,^9^,^10^,^11^. Second, the central cells at the prospective eyespot can induce ectopic fates in surrounding cells when transplanted into a different portion of the wing tissue^7^,^8^,^12^,^13^,^14^.

However, there is concern that butterfly wing eyespots are too large for a diffusible morphogen to form a stable gradient^15^,^16^,^17^. Colour pattern analysis of diverse nymphalid eyespots revealed that many nymphalid eyespots are not “typical” but are “deformed” in a way that the gradient model cannot explain logically^16^,^18^. Furthermore, physical damage experiments with *Junonia almana* revealed dynamic interactions between adjacent eyespot centres^11^. A striking finding is that when a large eyespot is damaged to become smaller, an adjacent small eyespot becomes larger, suggesting an inhibitory effect from the large eyespot to the small one^11^. Accordingly, as an alternative to the gradient model, the induction model has been proposed^19^,^20^, based on local self-activation and lateral inhibition^21^,^22^,^23^,^24^.

The validity of the induction model should be investigated further, but at present, more observational data are required to understand the developmental functions of eyespot organisers and differentiating epithelial cells in general in butterfly wing tissues. For this purpose, we have previously examined structures of pupal epithelial cells on the dorsal hindwings of the blue pansy butterfly *Junonia orithya*, using a real-time *in vivo* imaging system^25^,^26^. We have revealed vertically elongated processes of immature epithelial cells as deep as 130 μm in the dorsal hindwing^25^,^26^. Importantly, organisers for the border symmetry system (eyespot organizers) and for the marginal band system (edge spot organisers) are both indented on the surface of the dorsal hindwing^26^. That is, a cluster of epithelial cells forms a gentle cone-shaped hollow from the plane of the wing surface. Organising cells are likely to be located at the bottom of the indentation. Similar structures have been demonstrated in the dorsal forewing, and they are associated with the pupal cuticle focal spots^10^,^27^. Because of this three-dimensionality of the prospective eyespot region, we failed to directly examine epithelial cells at the bottom of the focal indentation; they were covered with thick cuticle, preventing them from being stained^25^,^26^.

However, it is still of great interest to directly observe the functioning organisers *in vivo* in the developing butterfly wing tissues. We reasoned that the hindwing eyespot organiser may be too large to stain the cells at the bottom of the focal indentation and that smaller eyespots may allow the staining and observation of the cells. In the present study, we focused on an anterior eyespot on the ventral forewing of *J. orithya* and successfully stained and observed the “focal cells” at the bottom of the focal indentation, using an *in vivo* observation system (Fig. 1A). Focal indentation of the ventral forewing is likely similar to that of the dorsal hindwing reported previously^26^. In the present study, comparisons were made at three regions of the ventral forewing: the focal, adjacent, and basal regions (Fig. 1B,C). The butterfly wing configuration is illustrated in Fig. 1D,E for convenience of reference. Together, this study presents important descriptive data on the morphology of organizing cells and developing epithelial cells in butterfly wings.

## Results

### Structure of the focal indentation

We double-stained epithelial cells with SYBR Green I for nuclei and MitoTracker Red for mitochondria. The overall structure of the focal indentation was revealed. The focal indentation was approximately 200–300 μm in diameter at the top surface but elongated slightly toward the proximal direction (*n* = 5) (Fig. 2A; Supplementary Video 1). In a representative sample, from an arbitrary zero point, the whole indentation extended from 20.91 μm at the top to 47.51 μm at the bottom (thus having a depth of 26.60 μm), assuming that mitochondria located at the apical side of a cell serve as an apical indicator. The depth of the indentation in five individuals was 24.64 ± 3.06 μm (mean ± SD) (*n* = 5). The bottom of the indentation was approximately 100 μm in diameter. Going more deeply from the bottom surface of the indentation, a small number of nuclei was clearly observed. A vertical cross section confirmed these observations (Fig. 2B).

Cells in and around the focal indentation were roughly classified into three categories based on their locations (Fig. 3A). Lip cells surrounded the indentation, forming a gentle slope. Cells that were located at the bottom of the indentation were called nadir cells because they are at the bottom when the ventral side is up. Between the lip cells and the nadir cells, there were peri-nadir cells. It appeared that there was a cluster of nadir cells at the bottom, and they were surrounded by four clusters of peri-nadir cells (Fig. 3B). The distal side of the nadir cells appeared to be a steep cluster of peri-nadir cells, although clarity of this feature varied among five individuals that were examined. We believe that the nadir cells function as organiser cells.

At the very bottom of the focal indentation, very few nuclei were observed (Fig. 3C). These nuclei were associated with mitochondria. Overall, mitochondria appeared to be aligned in the anteroposterior direction. The directional alignment of mitochondria probably indicates that they are present in the directional horizontal processes that connect two nadir cells. However, these mitochondrion-associated nuclei and aligned processes were not unique to the cluster of nadir cells. They were also observed in peri-nadir cells and lip cells in the focal region (Fig. 2; Supplementary Video 1) as well as in cells in the adjacent and basal regions (Supplementary Video 2).

### Nuclear and mitochondrial double staining of three regions

Here, we examined cellular structures of the three regions using the double staining for nuclei and mitochondria (Fig. 4). In the focal region, nuclei were mostly globular (sphere-like) (*N* = 10; *N* indicates the number of individuals examined) (Fig. 4A,B). Many mitochondria were distributed at the apical side, together forming an inverted cone shape. Similar features were observed in the cells of the adjacent region with globular nuclei, but flattened nuclei were also observed there (*N* = 8) (Fig. 4C). In contrast, in the basal region, most nuclei were flattened ovals and were tilted toward the distal side of the wing, and the distinction between the mitochondrial and nuclear layers was less clear (*N* = 5) (Fig. 4D,E). In all three regions, some nuclei were located very close to the apical surface, and others were located deeper than the mitochondrial layer. However, this distribution pattern of nuclei was more prominent in the focal region.

When the apical surface was set at 0 μm, the deepest tip of the mitochondrial inverted cone was observed at a depth of approximately 1–10 μm in all three regions. The deepest part of the mitochondrial layer was 3.51 ± 3.40 μm (mean ± SD) (*N* = 10, *n* = 221; *n* indicates the number of samples measured) for the focal region, 2.63 ± 1.47 μm (*N* = 8, *n* = 205) for the adjacent region, and 2.15 ± 0.94 μm (*N* = 5, *n* = 161) for the basal region (Fig. 4F). High SD value for the focal region originated from the fact that the mitochondrial layer was elongated deep, mainly in the focal region, resulting in higher depth variation (Fig. 4A). However, there was no significant difference in the depth of the mitochondrial layer (*p* = 0.98 for any pairs; Holm-corrected *t*-test). In all the three regions, mitochondria were detected not only at the apical inverted cones but also around nuclei and sparsely in other cytoplasmic locations.

### Whole-cell morphology

We stained epithelial cells with CFSE to observe the morphology of whole cells (Fig. 5). It appeared that cell size varied in the focal region (*N* = 6) (Fig. 5A,B) but not much in other regions (*N* = 4 in the adjacent region; *N* = 10 in the basal region) (Fig. 5C,D). As expected from the previous nuclear and mitochondrial double staining, cells in all three regions were large at the apical surface, and from the apical surface to portions approximately 5 μm deeper, cells became thinner. Below, many cells exhibited a swelling structure or “cell body” that likely contains a nucleus. This constriction-swelling structure was not found in the hindwing cells in the previous study^26^.

When the apical surface was set at 0 μm, the focal, adjacent, and basal regions had their deepest signals at 26.47 ± 3.59 μm (mean ± SD) (*N* = 6; *n* = 202), 26.31 ± 2.41 μm (*N* = 4; *n* = 107), and 25.76 ± 5.90 μm (*N*  = 10; *n* = 235), respectively (Fig. 5E). The distribution of the deepest signals exhibited no statistically significant difference among the three regions (*p* = 1.0 for any pairs; Holm-corrected *t*-test).

Angles of cell axis in relation to the apical tissue surface were also measured (Fig. 5D). Angles of the focal, adjacent, and basal regions were 88.64 ± 8.42 μm (*N* = 5; *n* = 44), 95.37 ± 8.97 μm (*N* = 4; *n* = 23), and 73.26 ± 14.25 μm (*N* = 9; *n* = 54), respectively (Fig. 5F). Thus, cells in the focal and adjacent regions were at nearly 90°, but the basal cells were relatively sharply angled, with significant differences from the focal and adjacent cells (*p *< 0.0001 for both pairs; Holm-corrected *t*-test).

Optical serial sections of cells were examined in three regions (*n* = 3 in each region) (Fig. 6; Supplementary Videos 3,4,5). Horizontal processes were present in all regions, which may be equivalent to epidermal feet in the hindwing^26^. Many processes were directionally aligned. The horizontal processes were most frequent at 4–8 μm in depth, but they were found down to approximately 10 μm in depth. In the focal and adjacent regions, relatively large endosome-like spherical structures were prominent (Fig. 6A,B), and these structures were also found in the hindwing^26^. Interestingly, the large endosome-like structures were not present in the basal region (Fig. 6C).

### Changes in nuclear volume over time

Because DNA synthesis for mitosis and also later for polyploidisation are known to take place in developing wing epithelium during the pupal stage^28^,^29^,^30^, the possibility of time-dependent changes in nuclear volume was examined using SYBR Green I (Fig. 7A). Doublet nuclei were observed in all regions but may be more frequent in the focal region (Fig. 7A). Nuclear volumes of the focal, adjacent, and basal regions at 1 h post-pupation were 275.08 ± 90.40 μm^3^ (mean ± SD) (*N* = 4; *n* = 221), 303.81 ± 90.12 μm^3^ (*N* = 4; *n* = 161), and 301.42 ± 74. 80 μm^3^ (*N* = 4; *n* = 205), respectively, which changed at 10 h post-pupation to 474.60 ± 110.04 μm^3^ (*N* = 3; *n* = 222), 454.65 ± 118.57 μm^3^ (*N* = 3; *n* = 356), and 437.64 ± 169.04 μm^3^ (*N* = 3; *n* = 143), respectively. Thus, nuclear volume increased significantly from 1 h to 10 h post-pupation in all three regions (*p *< 0.0001 for all 1 h–10 h pairs; Holm-corrected *t*-test) (Fig. 7B).

## Discussion

The present study successfully observed the cells at the bottom of the focal indentation. A similar attempt was previously made using the dorsal hindwing eyespots, but because of the thick cuticle overlay, clear staining was not possible^26^. Here, we used the ventral forewing anterior eyespot, which is much smaller than the hindwing eyespot that was examined previously. This simple approach, together with a careful staining procedure, solved the technical issue of staining organiser cells at the bottom of the focal indentation. To our knowledge, this is the first report to describe the organiser cells *in vivo* in butterfly wings.

The focal indentation was approximately 200–300 μm in diameter at the top surface and approximately 100 μm in diameter at the bottom, where relatively few nadir cells were found. The depth was approximately 25 μm, and the focal indentation thus forms a gentle slope. The mechanism by which the focal indentation is generated remains unclear, but it may have to do with cellular proliferation, apoptosis, growth, or morphological change at the cellular level because these cellular changes can cause physical torsion in the epithelial tissue, resulting in deformation of a planar surface. The biological significance of the focal indentation is obscure, but it may play an important role in eyespot formation because the size of the pupal cuticle focal spots (below which focal cells are located) is correlated with the adult eyespot size^10^,^27^. The epithelial distortion that is created by the focal indentation may be used as a physical signal to transmit morphogenic information.

In all three regions, cells were elongated in depth with an average length of 26 μm. This is much shorter than the hindwing cells that were reported previously^26^, which extended as deep as 130 μm. This difference may be inherent to a particular wing surface, but a more likely explanation would be that the developmental stages at the time of observation (1 h post-pupation) differ between the dorsal hindwing cells and the ventral forewing cells. During the pre-pupal stage, cells would vertically elongate, but then the dorsal and ventral epithelial sheets are attached to each other later in development. The hindwing cells likely develop a few steps ahead of the forewing cells, judging from the sensitivity to pharmacological injections^31^. Indeed, the hindwing nuclei appear to be larger, extending to 20 μm in depth, than the forewing ones. However, we cannot completely eliminate the possibility that the deeper portions of the forewing cells were not detected in this study due to unknown technical reasons.

Mean depth of mitochondrial layer was not statistically different among the three regions of interest, but the high SD value of the focal region is to be explained. This originated from the fact that the mitochondrial layer was elongated deep in the focal region. This deep-situated mitochondrial layer may be considered unique in the focal region, possibly indicating high metabolic activities in focal cells. In the CFSE staining, we observed a “cell body” below the mitochondrion-rich inverted cone. The cell body very likely contains a nucleus, based on the SYBR Green I staining. These cellular morphological features (i.e., the mitochondrial inverted cone and the cell body) were not detected in the dorsal hindwing cells^26^. Well-developed endosome-like structures in the focal region may also indicate high levels of metabolic activities. An additional indication of high metabolic activities is that the prospective eyespots likely have more tracheal branches than other regions^25^.

Morphology of nucleus differed among the three regions. It was almost globular in the focal region but a flattened oval in the basal regions. The adjacent region contained a mixture of flattened and globular nuclei. It has been known that after repeated nuclear divisions, nuclear morphology in the tobacco hornworm moth *Manduca sexta* changes from flattened oval to an almost globular shape^28^. Differences in developmental stages among the three regions could be one of the reasons for different nuclear morphology. Doublet nuclei were observed frequently in the focal region. Thus, the focal region may be more active in DNA amplification and cell division. An increase in nuclear volume from 1 h to 10 h post-pupation was significant in all three regions. Because nuclear volume directly reflects the amount of nuclear DNA^29^ and because mitotsis likely takes place during this time period^30^,^32^,^33^,^34^, this finding probably indicates active DNA synthesis for mitosis in these cells.

Horizontal processes, possibly epidermal feet or cytonemes that connect pairs of cells, were found in this study and in the previous study^26^. The nadir cells were vertically elongated but likely connected horizontally, mainly in the anteroposterior direction. These horizontal processes may contribute to the cellular cluster formation to unite vertical processes. Both in the forewing and in the hindwing, horizontal processes are present up to approximately 5 μm in depth. In both cases, these cellular processes contain mitochondria, suggesting a mechanism for intercellular transfer of mitochondria. In the hindwing, additional deep horizontal processes are present at 60–80 μm in depth^26^. Cell clustering through horizontal processes may be a general feature of butterfly wing epithelium, which would contribute to cellular communication. In *Drosophila*, cytonemes play a critical role in distributing morphogenic signal proteins^35^,^36^,^37^,^38^,^39^. It is possible that the horizontal processes that were observed in this study function as cytonemes to distribute morphogenic signal proteins. Although we did not examine calcium activity in the forewing, the horizontal processes may have a role in transmitting calcium signals (or other signals), as in the hindwing^40^.

**Figure 1:**
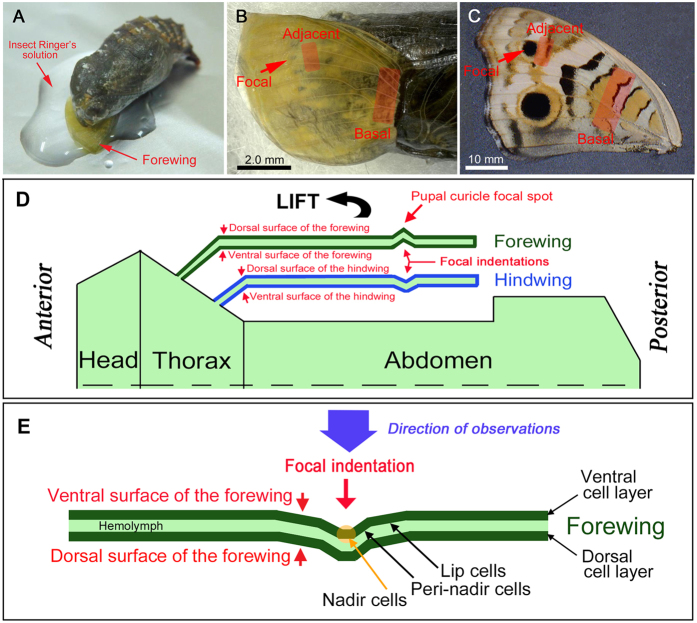
Pupal wing operations, three regions of observations, and schematic illustrations of the butterfly wing system. (**A)** An operated pupa. The right forewing was lifted and placed on a piece of cover glass. (**B**) A lifted forewing on the ventral side to be observed. Three regions of observations (the focal, adjacent, and basal regions) are indicated. (**C**) An adult ventral forewing. Three regions of observations corresponding to the pupal wing are indicated. (**D**) Schematic illustration of the butterfly wing configuration in a pupa. Wings are sacs of a single epithelial cell layer. Focal indentations and pupal cuticle focal spot are shown. For experiments, the forewing is lifted so that the ventral surface of the forewing is exposed. Only one side of a pupa is illustrated. (**E**) Schematic illustration of the pupal forewing. Focal indentation and the direction of observations are shown. The focal indentation consists of three different cells: nadir cells, peri-nadir cells, and lip cells. Individual cells are not illustrated.

**Figure 2:**
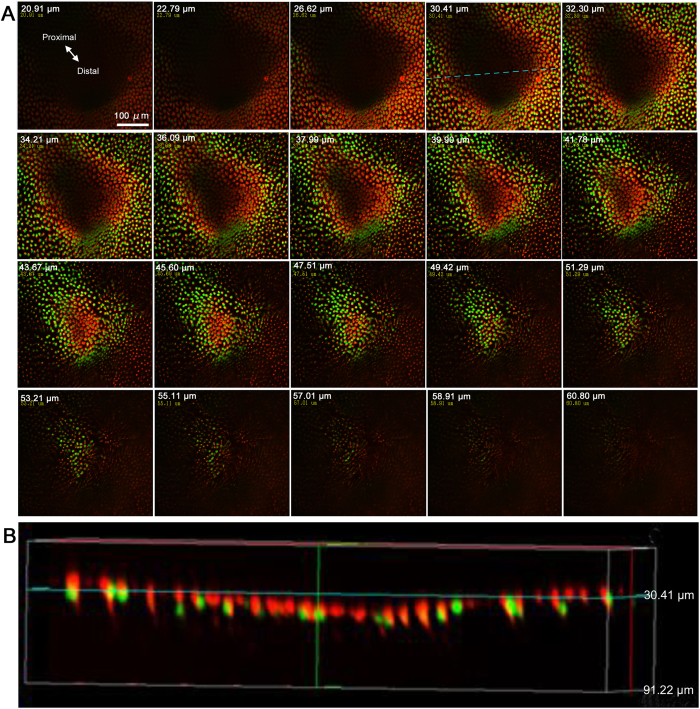
Focal indentation stained with SYBR Green I for nuclei and MitoTracker Red for mitochondria. (**A**) Horizontal optical sections. Sections are presented from the top left corner (shallow sections) to the bottom right corner (deep sections). Depth from the apical surface (0 μm point set arbitrarily) is shown in each panel. See also Supplementary Video 1. (**B**) Vertical optical section. The horizontal position of the vertical section is shown as a broken blue line in panel A, at 30.41 μm.

**Figure 3:**
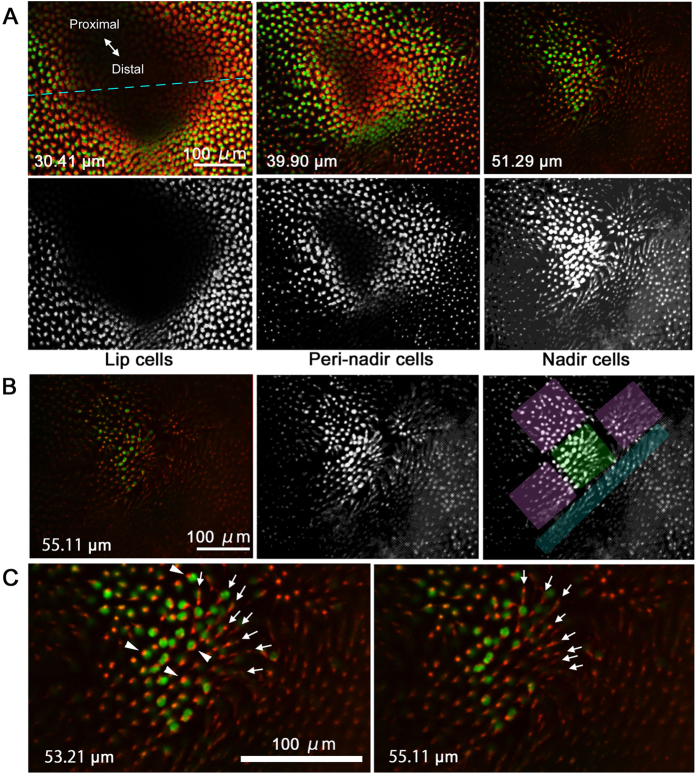
Detailed images of the focal indentation. Depth of the horizontal section is shown in each panel as in Fig. 2. (**A**) Three types of cells were identified based on their locations in the indentation: lip cells, peri-nadir cells, and nadir cells. The top three panels are identical to those in Fig. 2. The three bottom panels are half-tone sketches from the top panels. The broken blue line indicates the position of the vertical section shown in Fig. 2B. (**B**) Deepest nadir cells at the bottom of the focal indentation. The original image (left) was converted to half-tone sketches (middle and right). Possible cellular clusters are indicated in green (nadir cells), pink (peri-nadir cells), and blue (peri-nadir cells). (**C**) High magnification of the nadir cells. Red mitochondrial signals signify the possible horizontal processes. Directions of the horizontal processes are indicated by arrows. See also Supplementary Video 2 for comparison.

**Figure 4:**
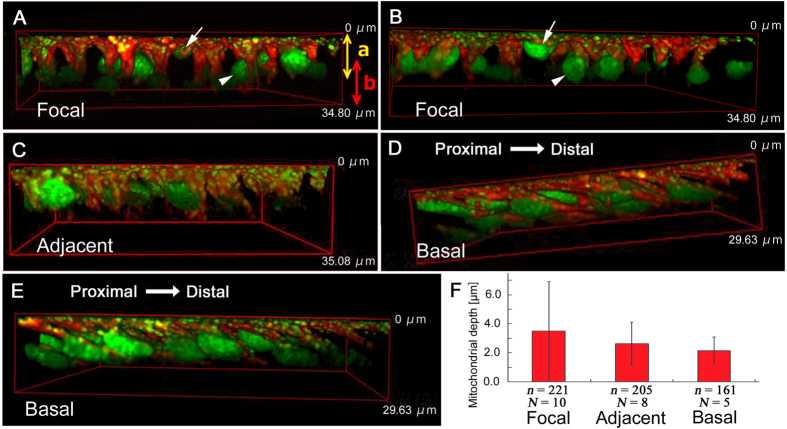
Three-dimensional reconstruction of wing epithelial cells double stained with SYBR Green I for nuclei and MitoTracker Red for mitochondria. Vertical length of the 3D cuboid is shown in each panel. (**A**,**B**) Focal region. The apical layer (indicated as “a”) contains most mitochondria and some nuclei (arrows), whereas the basal layer (indicated as “b”) contains some nuclei (arrowheads). The distinction between these two layers is solely for convenience, but these two layers are indistinguishable in the other regions. Mitochondria form a cluster of inverted cone in a cell. (**C**) Adjacent region. (**D**,**E**) Basal region. Cells are tilted toward the distal direction. (**F**) Mitochondrial depth (the deepest mitochondrial location) from the apical surface. Shown are mean values ± standard deviation. The number of cells examined (*n*) and the number of pupae used (*N*) are indicated.

**Figure 5:**
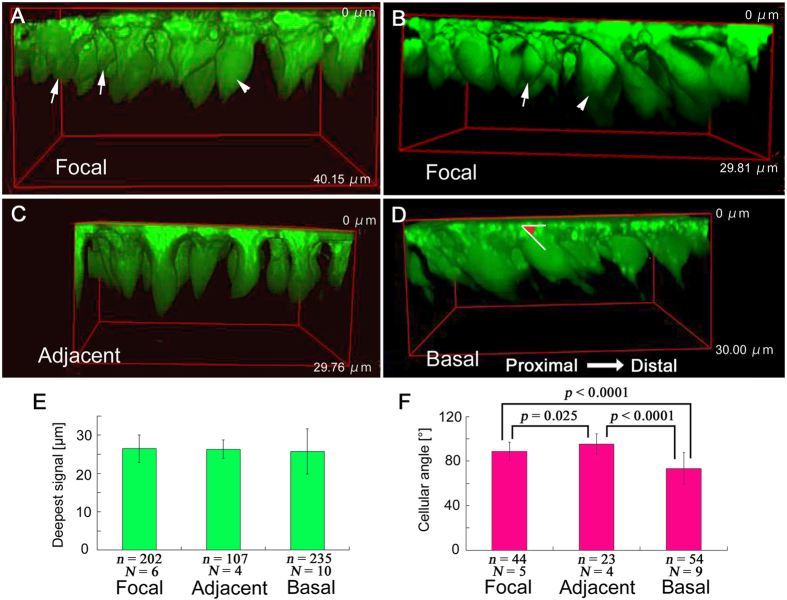
Three-dimensional reconstruction of wing epithelial cells stained with CFSE for cytoplasm. Vertical length of the 3D cuboid is shown in each panel. (**A**,**B**) Focal region. Relatively small cells (arrows) and relatively large cells (arrowheads) are indicated. (**C**) Adjacent region. Cells are relatively homogeneous. (**D**) Basal region. Cells are relatively homogeneous. An example of the measured cellular angle is indicated. (**E**) Deepest CFSE fluorescent signal from the apical surface. Shown are mean values ± standard deviation. The number of cells examined (*n*) and the number of pupae used (*N*) are indicated. (**F**) Cellular angle. Shown are mean values ± standard deviation. The number of cells examined (*n*) and the number of pupae used (*N*) are indicated.

**Figure 6:**
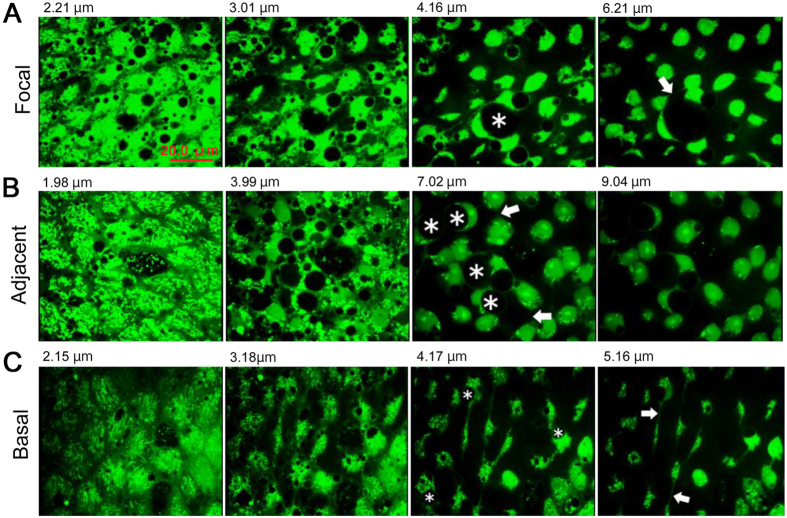
Serial optical sections of epithelial cells stained with CFSE. Panels are presented from the apical surface (left) to deeper layers (right). Depth from the apical surface is indicated in each panel. Endosome-like structures (asterisks) and horizontal processes that connect two cells (arrows) are indicated. (**A**) Focal region. See also Supplementary Video 3. (**B**) Adjacent region. See also Supplementary Video 4. (**C**) Basal region. See also Supplementary Video 5.

**Figure 7:**
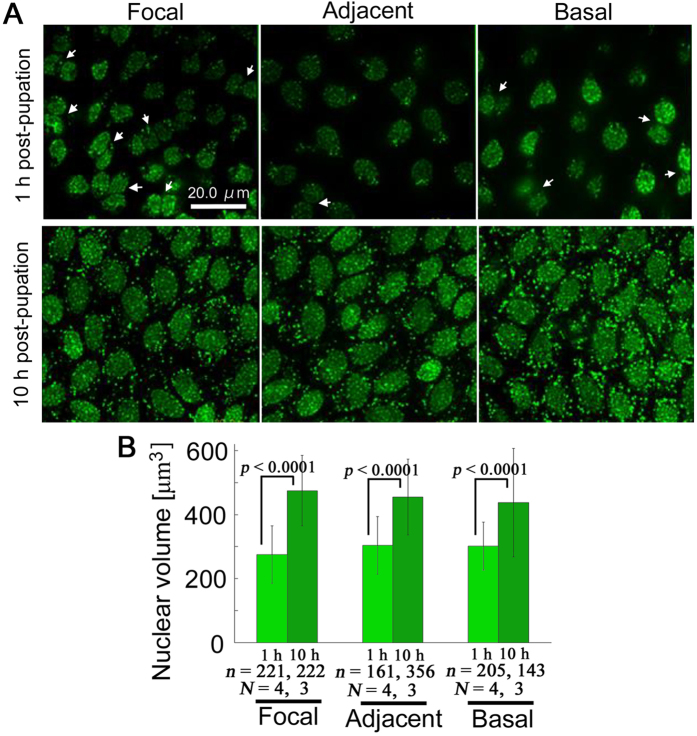
Comparison between 1 h and 10 h post-pupation. (**A**) SYBR Green I staining for nuclei. Two (or more) nuclei are associated together, especially in the focal region (arrows) 1 h post-pupation. Larger nuclei are densely packed at 10 h post-pupation. (**B**) Changes in nuclear volume. The number of cells examined (*n*) and the number of pupae used (*N*) are indicated.

Notably, the focal cells that were observed in this study do not produce the white spot that is often located at the centre of a nymphalid eyespot. There is no white spot (often called a “focus”) at the centre of this eyespot (Fig. 1C). Instead, the focal cells produce black scales. However, this fact does not undermine the function of their organizing activity. The function of an organiser for eyespot induction is different from its function for white-spot induction in nymphalid butterflies^41^.

In the present study, nuclear volume significantly increased from 1 h to 10 h post-pupation. This increase was observed in all the three regions, and the increase probably reflects DNA synthesis for mitosis of epithelial cells. DNA synthesis for mitotic divisions are probably followed by DNA synthesis for polyploidisation without cell division. Scale cells are known to be large polyploid cells surrounded by smaller epithelial cells^28^,^30^,^32^,^33^. This means that cell size variation gradually increases in the wing epithelium as developmental stage advances. Polyploidisation may be closely related with cell size, scale size, and scale colour, leading to the ploidy hypothesis for colour pattern determination^34^. In the present study, large and small epithelial cell specifications were not clear, but cell size variation was observed in the focal region. This result may suggest that the focal cells are a few steps ahead of cells in other regions in terms of developmental stage.

In summary, most striking difference was found in nuclear morphology; globular nuclei in the focal region and flattened nuclear in the other regions. Other possible differences included deep-situated mitochondrial layer, relatively large endosome-like structures, apically located nuclei, high cell size variation, and frequent doublet nuclei in the focal region. Overall, these features of nadir cells were not very distinguishable from cells in other regions and may be consequences of position-dependent heterochronic development of epithelial cells. That is, the focal cells may be developmentally advanced compared to the adjacent and basal cells. This heterochronic development may play an important role in colour pattern formation in butterflies in general, as shown in previous studies^42^,^43^,^44^,^45^.

## Methods

### Butterflies

Throughout this paper, the blue pansy butterfly *J. orithya* (Linnaeus, 1758) was used. We collected female adult individuals or larvae from Okinawa-jima Island or Ishigaki-jima Island, the Ryukyu Archipelago, Japan. Eggs were collected from the collected females. Larvae were fed their natural host plant *Plantago major* collected from the field in Okinawa-jima at ambient temperature, approximately 27 °C. No permissions were required to collect this butterfly from the wild, to rear this butterfly in the laboratory, and to perform experiments with this butterfly in Okinawa, Japan.

### Pupal operation and staining

Pupae were subjected to the forewing lift operation^25^,^26^,^34^,^40^,^45^ and staining treatment with fluorescent dyes at the same ambient temperature. The right pupal forewing was lifted up within 30 min post-pupation. We used three fluorescent dyes; CFSE (5- or 6-(N-succinimidyloxycarbonyl)fluorescein 3′,6′-diacetate) (Dojindo Molecular Technologies, Kumamoto, Japan) for cytoplasm, SYBR Green I (Takara Bio, Kusatsu, Shiga, Japan) for nucleic acids, and MitoTracker Red CMXRos (ThermoFisher Scientific, Waltham, MA, USA) for mitochondria. Fluorescent dyes were diluted with DMSO (dimethyl sulfoxide) and adjusted to the final concentration with insect Ringer’s solution (10.93 g NaCl, 1.57 g KCl, 0.83 g CaCl_2_•2H_2_O, and 0.83 g MgCl_2_•6H_2_O per liter). The final dye concentrations were 10 μM for CFSE and 1 μM for MitoTracker Red. For SYBR Green I, the original solution was diluted at 1:10,000. However, to observe the wing tissue 10 h post-pupation, the SYBR Green I original solution was diluted at 1:1,000.

The lifted forewing was placed over the staining solution (50 μL) on a piece of glass so that the tissue was evenly stained for 20–30 min at approximately 27 °C. Then, the staining solution was removed, and the tissue was washed with insect Ringer’s solution. The pupa was then placed on a piece of cover glass with a small amount of insect Ringer’s solution (Fig. 1A). Observations began at 1 h post-pupation. To observe the wing tissue 10 h post-pupation, pupae stained with SYBR Green I were placed in the dark at approximately 27 °C. Observations began at 10 h post-pupation.

### Fluorescent confocal imaging system and image analysis

We used a Nikon inverted epifluorescence microscope Eclipse Ti-U (Tokyo, Japan) equipped with a Nikon Epi-Fl Filter Cube GFP-B (EX480/40, DM505, and BA535/50) and a Hamamatsu Photonics ImagEM EM-CCD camera (Hamamatsu, Japan). This microscope hardware system was controlled with a Hamamatsu Photonics AQUACOSMOS/RATIO analysis system. Laser beams at 488 nm (CFSE and SYBR Green I) and 561 nm (MitoTracker Red) were used for fluorescent excitation. To produce 3D images, 2D images were taken every 0.2 μm from the apical surface (defined as 0 μm) down to 20–40 μm. To obtain nuclear volume, we employed the Sync Measure 3D function of ImageJ (v. 1.48)^46^ using the images stained with SYBR Green I. Mitochondrial DNA was also stained, but they were mostly distributed at the apical surface, above the nucleus. Thus, the mitochondrial layer was excluded to calculate the nuclear volume. Final images were processed using Adobe Photoshop Elements 11. Half-tone images were produced using the filtering and sketch function of this software.

### Statistical analysis

The statistical software R, version 3.2.1 (The R foundation for Statistical Computing, Vienna, Austria), was used to perform pairwise comparisons using *t*-tests with pooled standard deviation and with *p*-value adjustment with Holm’s method. Mean values reported in the text are ground mean values. To calculate the ground mean value, mean value of data samples for each individual pupa was first obtained, and the sum of these mean values was divided by the number of individuals (*N*). The numbers of samples (*n*) reported in the text are sum of the numbers of data samples used to obtain the ground mean value. However, all sample data were directly subjected to *t*-tests, because cell variation in any given individual was sufficiently large and because there was no bias among these individuals.

## Additional Information

**How to cite this article**: Iwasaki, M. *et al*. Butterfly eyespot organiser: *in vivo* imaging of the prospective focal cells in pupal wing tissues. *Sci. Rep.* **7**, 40705; doi: 10.1038/srep40705 (2017).

**Publisher's note:** Springer Nature remains neutral with regard to jurisdictional claims in published maps and institutional affiliations.

## Supplementary Material

Supplementary Video 1

Supplementary Video 2

Supplementary Video 3

Supplementary Video 4

Supplementary Video 5

Supplementary Information
