# Supplementary material for: Butterfly eyespot organiser: in vivo imaging of the prospective focal cells in pupal wing tissues
Source: Sci Rep. 2017 Jan 17;7:40705. doi: 10.1038/srep40705 (PMC5240560; doi:10.1038/srep40705)
Supplement: Supplementary Information [file srep40705-s6.pdf]

Scientific Reports

Supplementary Information

Butterfly eyespot organiser: *in vivo* imaging of the prospective focal cells in pupal wing tissues

Mayo Iwasaki, Yoshikazu Ohno & Joji M. Otaki

The BCPH Unit of Molecular Physiology, Department of Chemistry, Biology and Marine Science, University of the Ryukyus, Okinawa 903-0213, Japan

Correspondence and requests for materials should be addressed to J.M.O. (otaki@sci.u-ryukyu.ac.jp)

Supplementary Video 1
Supplementary Video 2
Supplementary Video 3
Supplementary Video 4
Supplementary Video 5

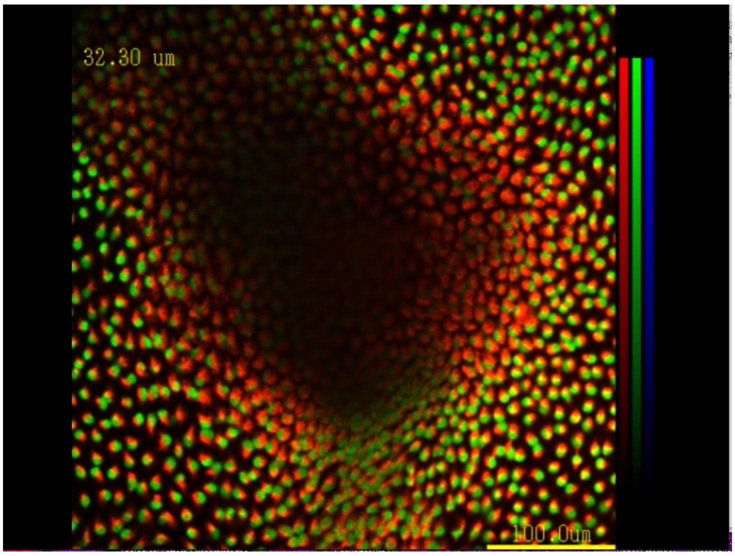

Supplementary Video 1. Focal indentation stained with SYBR Green I for nuclei and MitoTracker Red for mitochondria.

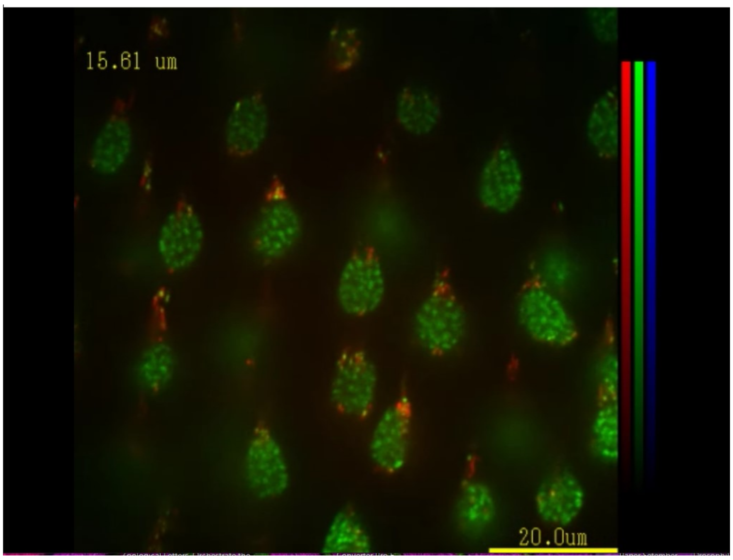

Supplementary Video 2. Basal region stained with SYBR Green I for nuclei and MitoTracker Red for mitochondria.

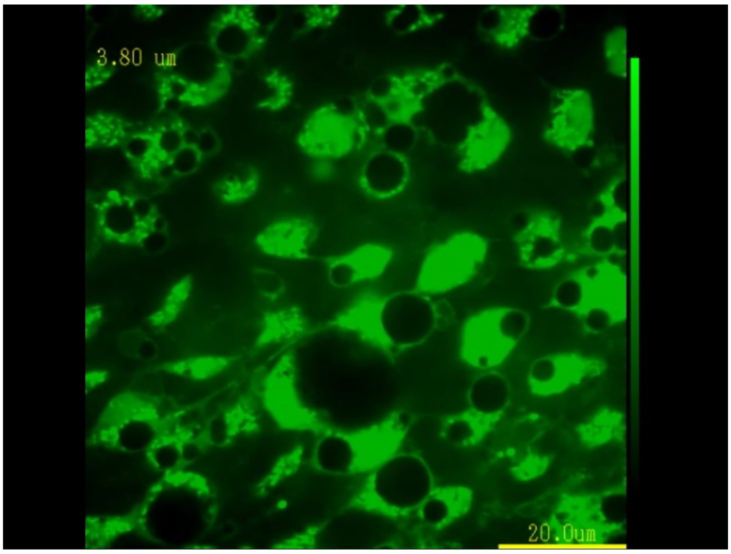

Supplementary Video 3. Focal region stained with CFSE for cytoplasm.

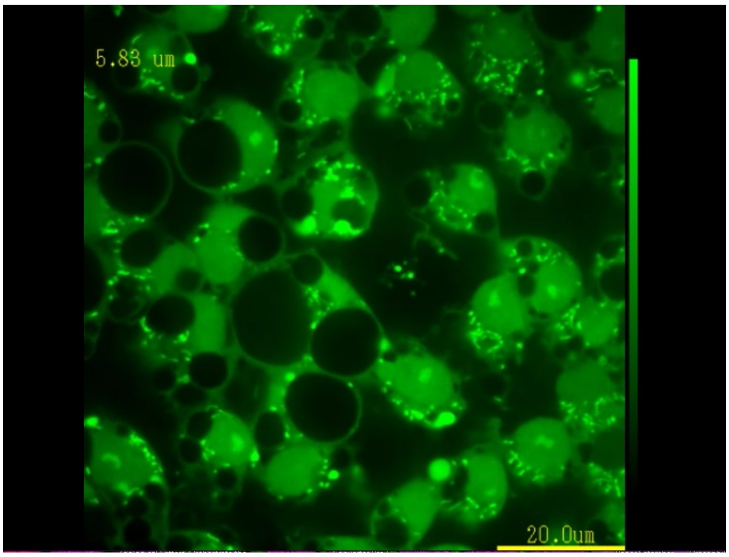

Supplementary Video 4. Adjacent region stained with CFSE for cytoplasm.

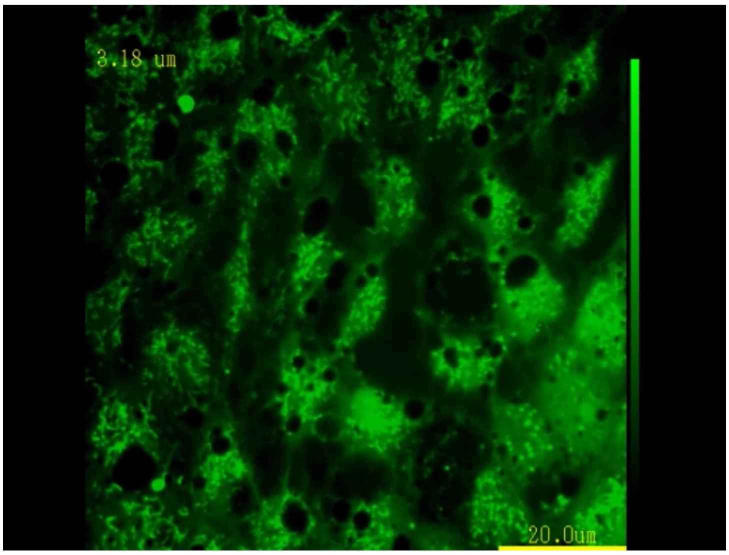

Supplementary Video 5. Basal region stained with CFSE for cytoplasm.
